# Assessing the equivalence of Web-based and paper-and-pencil questionnaires using differential item and test functioning (DIF and DTF) analysis: a case of the Four-Dimensional Symptom Questionnaire (4DSQ)

**DOI:** 10.1007/s11136-018-1816-5

**Published:** 2018-02-21

**Authors:** Berend Terluin, Evelien P. M. Brouwers, Miquelle A. G. Marchand, Henrica C. W. de Vet

**Affiliations:** 1 Department of General Practice and Elderly Care Medicine, Amsterdam Public Health Research Institute, VU University Medical Center, Amsterdam, The Netherlands; 2 Scientific Center for Care and Welfare (Tranzo), Tilburg University, Tilburg, The Netherlands; 3 CentERdata Institute for Data Collection and Research, Tilburg University, Tilburg, The Netherlands; 4 Department of Epidemiology & Biostatistics, Amsterdam Public Health Research Institute, VU University Medical Center, Amsterdam, The Netherlands

**Keywords:** Measurement equivalence, Web-based questionnaire, Paper-and-pencil questionnaire, Differential item functioning, Differential test functioning, Bifactor model

## Abstract

**Purpose:**

Many paper-and-pencil (P&P) questionnaires have been migrated to electronic platforms. Differential item and test functioning (DIF and DTF) analysis constitutes a superior research design to assess measurement equivalence across modes of administration. The purpose of this study was to demonstrate an item response theory (IRT)-based DIF and DTF analysis to assess the measurement equivalence of a Web-based version and the original P&P format of the Four-Dimensional Symptom Questionnaire (4DSQ), measuring distress, depression, anxiety, and somatization.

**Methods:**

The P&P group (*n* = 2031) and the Web group (*n* = 958) consisted of primary care psychology clients. Unidimensionality and local independence of the 4DSQ scales were examined using IRT and Yen’s Q3. Bifactor modeling was used to assess the scales’ essential unidimensionality. Measurement equivalence was assessed using IRT-based DIF analysis using a 3-stage approach: linking on the latent mean and variance, selection of anchor items, and DIF testing using the Wald test. DTF was evaluated by comparing expected scale scores as a function of the latent trait.

**Results:**

The 4DSQ scales proved to be essentially unidimensional in both modalities. Five items, belonging to the distress and somatization scales, displayed small amounts of DIF. DTF analysis revealed that the impact of DIF on the scale level was negligible.

**Conclusions:**

IRT-based DIF and DTF analysis is demonstrated as a way to assess the equivalence of Web-based and P&P questionnaire modalities. Data obtained with the Web-based 4DSQ are equivalent to data obtained with the P&P version.

**Electronic supplementary material:**

The online version of this article (10.1007/s11136-018-1816-5) contains supplementary material, which is available to authorized users.

## Introduction

Many questionnaires have been developed and validated as paper-and-pencil (P&P) questionnaires. However, over the past few decades, many of these questionnaires have increasingly been administered using electronic formats, in particular as Web-based questionnaires [1]. Advantages of data collection over the Internet include reduced administrative burden, prevention of item nonresponse, avoidance of data entry and coding errors, automatic application of skip patterns, and in many cases cost savings [1]. A Web-based questionnaire that has been adapted from a P&P instrument ought to produce data that are equivalent to the original P&P version [2]. Measurement equivalence means that a Web-based questionnaire measures the same construct in the same way as the original P&P questionnaire, and that, consequently, results obtained with a Web-based questionnaire can be interpreted in the same way as those obtained using the original P&P questionnaire. However, migration of a well-established P&P questionnaire to a Web-based platform does not guarantee that the Web-based instrument preserves the measurement properties of the original P&P questionnaire. Necessary modifications in layout, instructions, and sometimes item wording and response options might alter item response behavior. Therefore, it is recommended that measurement equivalence between a Web-based questionnaire and the original P&P questionnaire be supported by appropriate evidence [2]. Four reviews of such equivalence studies suggested that, in most instances, electronic questionnaires and P&P questionnaires produce equivalent results [3–6]. However, this is not always the case [4]. In this paper, we will demonstrate the use of modern psychometric methods to assess the equivalence across two modalities of a questionnaire. This is illustrated by analyzing the Web-based and P&P versions of the Four-Dimensional Symptom Questionnaire (4DSQ), a self-report questionnaire measuring distress, depression, anxiety, and somatization.

In 2009, the International Society for Pharmacoeconomics and Outcomes Research (ISPOR) electronic patient-reported outcomes (ePRO) Good Research Practices Task Force published recommendations on the evidence needed to support measurement equivalence between electronic and paper-based patient-reported outcome (PRO) measures [2]. The task force specifically recommended two types of study designs, the randomized parallel groups design and the randomized crossover design. In the former design, participants are randomly assigned to one of two study arms in which they complete either the P&P PRO or the corresponding ePRO. Mean scores can then be compared between groups. This is a fairly weak design to assess measurement equivalence [7]. In the latter design, participants are randomly assigned to one of two study arms in which they either first complete the P&P PRO and then the ePRO or the other way around. Then, in addition to comparing mean scores, the correlation between the P&P score and the ePRO score can be calculated. The correlation, however, is little informative about the true extent of equivalence because of measurement error and retest effects. Measurement error attenuates the correlation, making it difficult to assess the true equivalence. Retest effects may further aggravate the problem. Retest effects are thought to be due to memory effects and specific item features eliciting the same response in repeated measurements [8]. Retest effects are assumed to diminish with longer intervals between measurements. However, longer intervals carry the risk of the construct of interest changing in between measurements, leading to underestimation of the true correlation.

The research designs, discussed above, assess only a small aspect of true measurement equivalence because they fail to address equivalence of item-level responses [7, 8]. Contemporary approaches to measurement equivalence employ differential item functioning (DIF) analysis [9]. Addressing equivalence of item-level information, DIF analysis has been used extensively to assess measurement equivalence across different age, gender, education, or ethnicity groups (e.g., [10]), or to assess the equivalence of different translations of a questionnaire (e.g., [11]). Whereas DIF analysis dates back to at least the 1980s [12], the method is relatively new in mode of administration equivalence research. In a non-systematic search, we identified only a dozen such studies (e.g., [7, 8, 13–15]). The ISPOR ePRO good research practice task force report briefly mentioned DIF analysis as ‘another approach’ without giving the method much attention [2]. The recent meta-analysis by Rutherford et al. did not include any studies using DIF analysis [6].

The idea behind DIF analysis is that responses to the items of a questionnaire reflect the underlying dimension (or latent trait) that the questionnaire intends to measure, and that two versions of a questionnaire are equivalent when the corresponding items demonstrate the same item-trait relationships. There are various approaches to DIF analysis including non-parametric (Mantel–Haenszel/standardization) [16, 17] and parametric approaches (ordinal logistic regression, item response theory, and structural equation modeling) [18–20]. In the present paper, we demonstrate the use of DIF analysis within the item response theory (IRT) framework by assessing measurement equivalence across a Web-based and the original P&P versions of the Four-Dimensional Symptom Questionnaire (4DSQ).

## Methods

### Study samples and design

DIF analysis compares the measurement properties of an instrument across two groups, usually referred to as ‘reference group’ and ‘focal group.’ In the present study, both groups consisted of clients, aged 18–80, from primary care psychology practices. The reference group, in which P&P 4DSQ data had been collected between December 2002 and February 2013, consisted of 2031 clients from a single large practice, whereas the focal group, in which Web-based 4DSQ data had been collected between April 2011 and September 2017, comprised 958 clients from 21 practices. In both groups, the data had been collected in the context of routine care.

### Measure

The Four-Dimensional Symptom Questionnaire (4DSQ) is a 50-item self-report questionnaire measuring the four most common dimensions of psychopathological and psychosomatic symptoms in primary care settings (see Online Resource 1) [21]. The distress scale (16 items) aims to measure the kind of symptoms people experience when they are ‘stressed’ as a result of high demands, psychosocial difficulties, daily hassles, life events, or traumatic experiences [22]. The depression scale (six items) measures symptoms that are relatively specific to depressive disorder, notably, anhedonia and negative cognitions [23–25]. The anxiety scale (12 items) measures symptoms that are relatively specific to anxiety disorder [25–27]. The somatization scale (16 items) measures symptoms of somatic distress and somatoform disorder [28, 29]. The 4DSQ employs a time-frame reference of 7 days. The items are answered on a 5-point frequency scale from ‘no’ to ‘very often or constantly.’ In order to calculate sum scores, the responses are coded on a 3-point scale: ‘no’ (0 points), ‘sometimes’ (1 point), ‘regularly,’ ‘often,’ and ‘very often or constantly’ (2 points) [21]. Collapsing the highest response categories ensures that relatively more weight is put on the number of symptoms experienced than on their perceived frequency. It also prevents the occurrence of sparsely filled, or even empty, response categories, which might cause estimation problems with various statistical procedures.

The four-dimensional factor structure of the 4DSQ has been confirmed in different samples [21, 30]. However, as the focus of the present study was on the measurement properties of the separate 4DSQ scales, all analyses were conducted scale-wise, ignoring relationships between the scales. The 4DSQ is freely available for non-commercial use at http://www.4dsq.eu.

### Statistical analysis

#### Initial analyses

We calculated basic descriptive statistics for the groups including gender composition, mean age and standard deviation (SD), and mean 4DSQ scale scores and SDs.

Because some calculations (e.g., model fit) require complete data, we applied single imputation of missing item scores using the ‘response function’ method that takes into account both differences between respondents and differences between items [31]. The method is superior to less sophisticated methods in recovering the properties of the original complete dataset [32].

#### Dimensionality and local independence

IRT requires that response data fulfill the assumptions of unidimensionality and local independence [33]. Unidimensionality refers to a scale’s item responses being driven by a single factor, i.e., the latent trait that the scale purports to measure. Strict unidimensionality, implying that only the intended dimension underlies the item responses and no other additional dimensions affect these responses, is rare in psychological measurements [34]. However, ‘essential unidimensionality’ will suffice as long as there is one dominant dimension, whereas other, weaker, dimensions do not impact the item scores too much [35]. Local independence means that responses to one item should be independent from responses to the other items of a scale, conditional on the dimension that the items and the scale purport to measure [36]. Local item dependence (LID) actually results from one or more additional dimensions (beyond the intended dimension) operating on the item responses. Therefore, LID analysis can be used to assess the dimensionality of a scale.

For each scale, we examined its dimensionality by first fitting a unidimensional IRT graded response model. Model fit was assessed by the M2* statistic for polytomous data [37] and various fit indices. Relatively good fit is indicated by the following fit indices: Tucker-Lewis index (TLI) > 0.95, comparative fit index (CFI) > 0.95, standardized root mean square residual (SRMSR) < 0.08, and root mean squared error of approximation (RMSEA) < 0.06 [38]. Note that these benchmarks were developed in the context of structural equation modeling, and that their validity in the context of IRT is not well known. On the other hand, measurement models in IRT and structural equation modeling are formally equivalent [39]. LID was assessed using Yen’s Q3 statistic [40]. This statistic represents the correlation between the residuals of two items of a scale after partialling out the dimension (or dimensions in case of a multidimensional model) that the scale purports to measure. The Q3 is not expected to be zero in the absence of LID. Due to ‘part-whole contamination,’ the expected Q3 proves to be slightly negative [41]. As proposed by Christensen et al., [42] we calculated critical Q3-values by parametric bootstrapping. For each group, for each scale, and for each (uni- or multidimensional) IRT model, we simulated 200 locally independent response data sets based on the item parameters and theta score distribution(s) obtained for a specified IRT model. We recorded the maximum Q3 for each dataset. Across the simulated datasets per group, per scale, and per model, we denoted the 99th percentile of the maximum Q3-values as the critical Q3-value. Observed Q3-values greater than this critical Q3-value were taken as indicating LID.

In order to assess the extent to which the scales can be considered to be essentially unidimensional, we build ‘bifactor’ models based on the LID information [34]. Bifactor models are characterized by one large general factor, underlying all items of a scale and measuring the intended construct of the scale, and one or more smaller specific factors underlying subsets of items [43]. Every item must load on the general factor and may load on one specific factor. In an iterative process, we tried to capture the LID by defining specific factors affecting items with the largest Q3-values in excess of the critical Q3-value [42]. After defining a specific factor, the bifactor model was assessed for model fit and Q3-values and the critical Q3 of that model were reassessed. Remaining LID was handled by assigning item pairs with LID to a new or existing specific factor until the LID was completely resolved, model fit deteriorated instead of improved, or standardized factor loadings < 0.2 emerged (standardized factor loadings < 0.2 represent less than 4% shared variance between the item and the factor). Importantly, our interest was in the ‘purified’ general factors and not in the minor specific factors.

In order to assess whether the scales were essentially unidimensional, we calculated the proportion of uncontaminated correlations (PUC) and the explained common variance (ECV), based on the best fitting bifactor models [44]. The PUC is an index of the data structure, i.e., an index of how many inter-item correlations are accounted for by the general factor only. Consider a 10-item scale and a bifactor model with 1 specific factor loading on four items. There are (10 × 9)/2 = 45 unique inter-item correlations among ten items. Within the specific factor, there are (4 × 3)/2 = 6 unique correlations. So, 6 out of 45 correlations among the items are confounded by the specific factor. Thus, the PUC is (45–6)/45 = 0.87. PUC values greater than 0.80 indicate low risk of bias when a multidimensional scale is treated as unidimensional [45]. The ECV is the common variance explained by the general factor divided by the total common variance of a scale, and represents an index of the relative strength of the general factor to the specific factors. ECV values greater than 0.70 are usually indicative of essential unidimensionality [46]. As an illustration of the bias caused by forcing multidimensional scales into a unidimensional model, we compared 2 theta estimations, one derived from the initial unidimensional IRT model ignoring LID, and another derived from the general factor of the best fitting bifactor model. Intra-individual theta differences greater than 0.2 or 0.5 logits (the metric of the theta scale) represent small or moderate differences in terms of effect size [47]. In addition, the Pearson correlation between the estimations was calculated.

#### Reliability

We calculated Cronbach’s alpha as a measure of internal consistency reliability. In addition, we calculated omega-total and omega-hierarchical coefficients based on the standardized factor loadings from the final bifactor models [43]. Omega-hierarchical can be regarded as an indicator of the strength of the general factor, and as such as a benchmark of essential unidimensionality [45].

#### Differential item functioning (DIF)

**Fig. 1:**
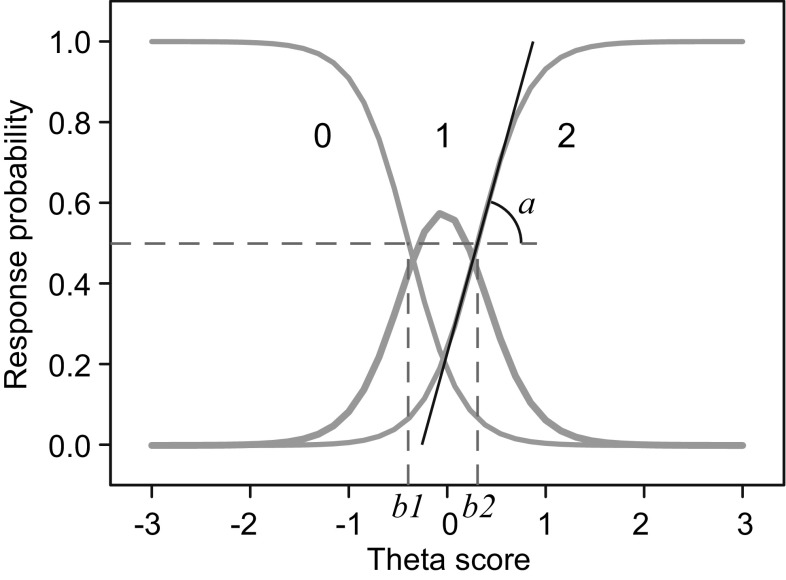
Item characteristic curve of an item with three ordered response categories (0, 1, and 2). Item parameters *a* (discrimination), *b*1 and *b*2 (difficulties) are indicated

We used DIF analysis in the IRT context, in which the probability of endorsing an item response category is modeled as a function of certain item characteristics and the trait levels of respondents [48, 49]. In the graded response model for polytomous items, the relationship between items and the underlying trait is defined by two types of item parameters, called ‘difficulty’ and ‘discrimination.’ The item-trait relationship can be graphically displayed by the item characteristic curve (Fig. 1). A polytomous item with 3 response categories is defined by two difficulty parameters (denoted *b*1 and *b*2) and 1 discrimination parameter (denoted *a*). The difficulty parameters (*b*1 or *b*2) are defined by the latent trait (theta) levels indicating the thresholds between response options. For the 4DSQ scales, *b*1 is located between category 0 and the two higher categories, while *b*2 is located between the lower categories and category 2 (see Fig. 1). The discrimination parameter *a* is defined by the slope of the item characteristic curve (at the thresholds *b*1 and *b*2), representing the item’s ability to discriminate between respondents standing low and high on the trait. Two items (or two versions of the same item) are deemed equivalent when they have the same relationships with the underlying trait, that is, when the items have similar item characteristics (difficulties and discriminations). For more detailed information about IRT, we refer to some excellent introductory papers, which are freely accessible on the Internet [48, 49].

DIF analysis in the IRT framework thus implies testing the equivalence of the item parameters of the corresponding items across two groups, the focal and reference group. This can be done using the Wald test, after appropriately linking the groups, i.e., placing all subjects on a common metric. Linking is usually accomplished by ‘anchor’ items with known invariance across the groups. However, in the absence of pre-specified anchor items, we followed a 3-stage procedure to first select appropriate anchor items and then testing items for DIF [50, 51]. In each stage, a multi-group unidimensional IRT graded response model was fitted to each scale in turn. The first stage constrained the item parameters to be the same across both groups to estimate the latent mean and variance of the focal group relative to the reference group. The second stage then provided preliminary linking between the groups by treating the estimated latent mean and variance as fixed, allowing the item parameters to be freely estimated and preliminarily tested for DIF. This stage was used to select items without DIF (*p* > 0.05) as anchor items. The third stage used the anchor items to link the groups, allowing means and variances freely estimated and the non-anchor items tested for DIF. Items with (Bonferroni corrected) *p* values < 0.001 and unsigned item difference in the sample (UIDS) values (see next section) effect sizes > 0.1 were deemed to have DIF. A UIDS of 0.1 is comparable with a standardized mean difference in item score of 5% of the item range (which is two points) [17].

To assess the severity of DIF, a final IRT model was fitted in which the parameters of the DIF items were freely estimated, while the parameters of the non-DIF items were constrained to be the same across both groups. The magnitude of DIF was then expressed as effect sizes based on expected item scores calculated twice for each member of the focal group, based on either the item parameters of the reference group or the item parameters of the focal group [52]. The signed item difference in the sample (SIDS) represents the mean difference in expected item scores in the focal group. The unsigned item difference in the sample (UIDS) represents the mean of the absolute difference in expected item scores in the focal group. Unlike the SIDS, the UIDS does not allow for cancelation of differences across respondents. The SIDS and UIDS are expressed in the metric of the scale score. On the other hand, the expected score standardized difference (ESSD) represents the Cohen’s *d* version of the SIDS. ESSD values > 0.2 can be interpreted as representing a small effect and > 0.5 as representing a moderate effect of DIF.

#### Differential test functioning (DTF)

Differential test functioning (DTF), the scale-level impact of item-level DIF, was expressed by a number of effect size measures based on expected scale scores [52]. The signed test difference in the sample (STDS) represents the sum of all SIDSs across all items of a scale, and allows for cancelation of differences in expected scores across items and persons. The unsigned test difference in the sample (UTDS) represents the sum of all UIDSs across all items of a scale. The UTDS allows no cancelation across items or persons. The unsigned expected test score difference in the sample (UETSDS) represents the average of absolute values of the expected test score differences in persons. The UETSDS reflects the true behavior of DIF on observed scale scores as it allows for cancelation across items but not across persons. The expected test score standardized difference (ETSSD) is the Cohen’s *d* version of the STDS.

#### Software

SPSS version 22 was used to prepare the data, impute missing item scores, and calculate mean scores and Cronbach’s alphas. All other analyses were conducted using the package ‘mirt’ version 1.21 for multidimensional item response theory [53, 54] within the statistical software R 3.2.5 [55].

## Results

### Initial analyses

The groups were reasonably comparable with respect to gender composition, mean age, and 4DSQ scores (Table 1). Thirty-four clients in the P&P group (1.7%) and six clients in the Web group (0.6%) had one or more missing item scores, which were imputed.

**Table 1:** Characteristics of the study participants by mode of administration group

Characteristics	Web group *n* = 958	P&P group *n* = 2031
Gender (% female)	58%	61%
Age, mean (SD)	42.9 (11.8)	40.1 (13.2)
4DSQ distress (range 0–32), mean (SD)	18.5 (8.6)	19.1 (8.2)
4DSQ depression (range 0–12), mean (SD)	3.3 (3.5)	3.7 (3.6)
4DSQ anxiety (range 0–24), mean (SD)	5.6 (5.4)	5.5 (5.1)
4DSQ somatization (range 0–32), mean (SD)	11.6 (7.1)	10.2 (6.6)

*4DSQ* Four-Dimensional Symptom Questionnaire, *SD* standard deviation; *Web* Web-based administration; *P&P* paper-and-pencil administration

### Unidimensionality and local independence

For every 4DSQ scale and for every group, Table 2 shows the fit indices of two models, the initial unidimensional model, and the final bifactor model. The M2* statistic follows a Chi-square distribution and, like the Chi-square statistic, is sensitive to large sample sizes. The fit indices suggested that the distress and somatization scales were not strictly unidimensional in both the P&P and the Web groups. On the other hand, the fit indices suggested relatively good fit of the unidimensional models of the depression and anxiety scales. Nevertheless, in all instances, some Q3-values suggested the presence of LID. The LID could not be resolved completely due to deterioration of model fit in case of the distress scale (in both groups), the depression scale (in the P&P group), and the anxiety scale (in the Web group). Standardized factor loadings < 0.2 occurred in case of the somatization scale (in both groups) and the anxiety scale (in the P&P group). The bifactor models demonstrated relatively good fit in all cases. The standardized factor loadings (provided in Online Resource 2) showed that the factor loadings of the unidimensional factors and the loadings of the general factors of the bifactor models of the same scales were very similar, suggesting that the unidimensional models predominantly represented the general factors [34]. The ECV values varied between 0.615 and 0.940, and the PUC values between 0.894 and 0.975, suggesting essential unidimensionality (Table 3). The theta estimations based on the unidimensional models did not differ much from those based on the general factors of the corresponding bifactor models. For the distress, depression, and anxiety scales, the theta differences were negligible (< 0.2) in more than 95% of the participants. Regarding the somatization scale, theta differences were somewhat larger: theta differences > 0.2 (small effect size) occurred in about 30% of the participants, but theta differences > 0.5 (moderate effect size) occurred in less than 2%. The presence of minor specific factors did not cause important bias in the estimation of the trait scores when these factors are ignored and the data are forced into unidimensional IRT models.

**Table 2:** Model fit indices and Q3-values for the initial unidimensional models and the final bifactor models by mode of administration group and 4DSQ scale

Scale	Group	Model	M2*	d*f*	*p*	TLI	CFI	SRMSR	RMSEA	90% CI of RMSEA	Q3_max_	Q3_crit_
Distress	P&P	Uni	1907.17	88	0.000	0.910	0.924	0.073	0.101	0.097–0.105	0.614	0.053
		Bif	546.14	85	0.000	0.976	0.981	0.044	0.052	0.048–0.056	0.230	0.074
	Web	Uni	1077.98	88	0.000	0.916	0.929	0.076	0.108	0.103–0.114	0.665	0.078
		Bif	373.91	85	0.000	0.975	0.979	0.048	0.060	0.053–0.066	0.262	0.103
Depression	P&P	Uni	24.46	3	0.000	0.983	0.994	0.046	0.059	0.039–0.082	0.085	− 0.024
		Bif	5.96	2	0.051	0.995	0.999	0.038	0.031	0.000–0.062	0.074	− 0.016
	Web	Uni	18.68	3	0.000	0.977	0.992	0.058	0.074	0.044–0.108	0.249	− 0.005
		Bif	4.04	2	0.132	0.995	0.999	0.034	0.033	0.000–0.079	0.074	0.084
Anxiety	P&P	Uni	190.35	42	0.000	0.979	0.984	0.049	0.042	0.036–0.048	0.145	0.059
		Bif	103.71	37	0.000	0.990	0.993	0.041	0.030	0.023–0.037	0.076	0.071
	Web	Uni	89.46	42	0.000	0.990	0.992	0.046	0.034	0.024–0.044	0.117	0.090
		Bif	73.63	39	0.001	0.992	0.994	0.043	0.030	0.020–0.041	0.107	0.093
Somatization	P&P	Uni	1316.19	88	0.000	0.870	0.890	0.072	0.083	0.079–0.087	0.350	0.056
		Bif	311.04	79	0.000	0.973	0.979	0.032	0.034	0.038–0.043	0.090	0.060
	Web	Uni	685.97	88	0.000	0.908	0.922	0.077	0.084	0.078–0.090	0.414	0.086
		Bif	326.80	81	0.000	0.959	0.968	0.048	0.056	0.050–0.063	0.147	0.100

Group: *Web* Web-based administration, *P&P* paper-and-pencil administration; model: *Uni* unidimensional model, *Bif* final bifactor model; M2* M2* statistic for polytomous data; *TLI* Tucker-Lewis index; *CFI* comparative fit index; *SRMSR* standardized root mean square residual; *RMSEA* root mean squared error of approximation; *90% CI* 90% confidence interval; *Q3*_*max*_ maximum Q3-value in the sample for the model; *Q3*_*crit*_ critical Q3-value for the model

**Table 3:** Indicators of scale dimensionality (PUC and ECV) and the difference between and correlations of theta estimations based on the unidimensional models and the general factors of the bifactor models

Scale	Group	PUC	ECV	Mean theta difference	99% range of differences	Correlation
Distress	P&P	0.975	0.756	0.002	− 0.211; 0.218	0.996
	Web	0.975	0.788	0.001	− 0.216; 0.229	0.996
Depression	P&P	0.933	0.940	− 0.008	− 0.069; 0.098	0.999
	Web	0.933	0.924	− 0.016	− 0.306; 0.291	0.996
Anxiety	P&P	0.894	0.823	− 0.017	− 0.226; 0.266	0.997
	Web	0.955	0.929	− 0.008	− 0.162; 0.182	0.999
Somatization	P&P	0.900	0.615	− 0.003	− 0.484; 0.560	0.974
	Web	0.942	0.688	− 0.007	− 0.425; 0.399	0.982

Group: *P&P* paper-and-pencil administration, *Web* Web-based administration; *PUC* percentage of uncontaminated correlations; *ECV* explained common variance

### Reliability

Table 4 presents an overview of the reliability estimates of the 4DSQ scales in both groups. Cronbach’s alpha and omega-hierarchical were greater than 0.80 and omega-total was greater than 0.90 for all scales in both groups. The omega ratios indicated that 88.7–97.9% of the total reliable scale score variance was accounted for by the general factor. This underlined the 4DSQ scales’ essential unidimensionality.

**Table 4:** Reliability of the 4DSQ scales by mode of administration group

Scale	Group	Cronbach’s alpha	Omega-h	Omega-total	Omega ratio
Distress	P&P	0.907	0.924	0.963	0.959
	Web	0.921	0.937	0.970	0.966
Depression	P&P	0.879	0.936	0.956	0.979
	Web	0.891	0.942	0.968	0.973
Anxiety	P&P	0.844	0.879	0.932	0.943
	Web	0.877	0.928	0.946	0.981
Somatization	P&P	0.833	0.819	0.923	0.887
	Web	0.866	0.870	0.938	0.928

*Omega-h* omega-hierarchical; *Omega ratio* omega-hierarchical/omega-total; *Web* Web-based administration; *P&P* paper-and-pencil administration

### Differential item functioning

After linking the groups on the latent mean and variance, suitable anchor items were identified for distress (seven items), depression (three items), anxiety (three items), and somatization (ten items). Ultimately, DIF was identified in three distress items and two somatization items (Table 5). All DIF was negative, indicating that the Web group tended to score a little lower on distress and somatization (conditional on the latent trait) due to the fact that the DIF items represented relatively somewhat more severe symptoms in the Web-based format (item parameters are provided in Online Resource 3). However, in terms of effect size (ESSD) the effect of DIF was small. For instance, the SIDS value of − 0.144 for ‘nausea or an upset stomach’ means that respondents to a Web-based 4DSQ scored on average 0.144 points lower on that item than respondents to a P&P 4DSQ with similar levels of somatization would do.

**Table 5:** Items with differential item functioning (DIF)

Scale	Item	Short item description	Wald (d*f* = 3)	*p*	SIDS	UIDS	ESSD
Distress	#17	feeling down or depressed	45.487	0.000	− 0.117	0.117	− 0.212
	#47	fleeting images of upsetting events	25.491	0.000	− 0.127	0.127	− 0.348
	#48	put aside thoughts about upsetting events	28.325	0.000	− 0.132	0.132	− 0.314
Somatization	#12	nausea or an upset stomach	42.245	0.000	− 0.144	0.148	− 0.308
	#13	pain in the abdomen or stomach area	23.506	0.000	− 0.104	0.104	− 0.238

*SIDS* signed item difference in the sample; *UIDS* unsigned item difference in the sample; *ESSD* expected score standardized difference

### Differential test functioning (DTF)

**Fig. 2:**
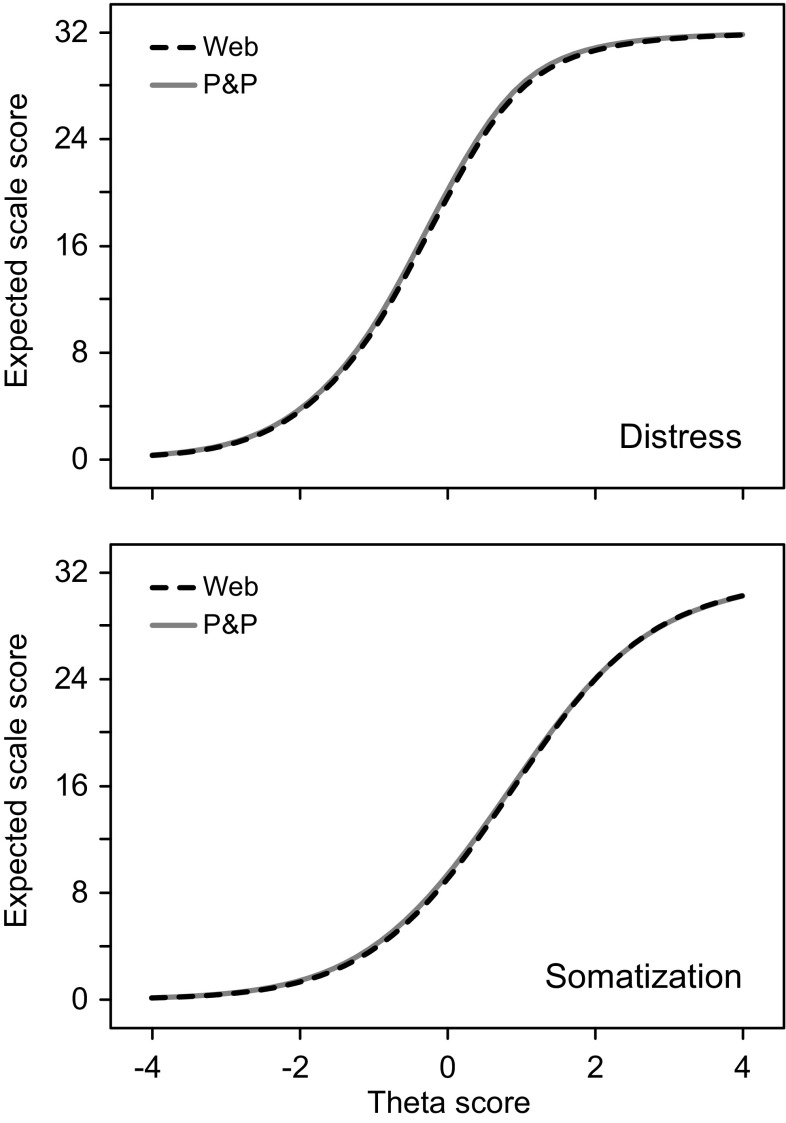
Differential test functioning: expected 4DSQ scale scores as a function of trait level (theta) by mode of administration group (*Web* Web-based administration, *P&P* paper-and-pencil administration)

Table 6 reveals that the impact of DIF on the distress and somatization scores was small in terms of mean difference in expected test scores across items and persons (STDS) and negligible in terms of Cohen’s *d* (ETSSD). Figure 2 displays the expected scale scores as a function of the DIF-free theta score by group, showing that the 4DSQ scale scores obtained by means of a Web-based questionnaire demonstrated similar relationships with the trait scores (thetas) as the 4DSQ scale scores obtained by means of a P&P questionnaire.

**Table 6:** Differential test functioning (DTF)

Scale	STDS	UTDS	UETSDS	ETSSD
Distress	− 0.377	0.377	0.377	− 0.046
Somatization	− 0.247	0.251	0.248	− 0.039

*STDS* signed test difference in the sample; *UTDS* unsigned test difference in the sample; *UETSDS* unsigned expected test score difference in the sample; *ETSSD* expected test score standardized difference

## Discussion

We examined measurement equivalence of the 4DSQ across the traditional P&P version and a modern Web-based version using DIF and DTF analysis. We identified DIF in five items from two scales. In terms of effect size, the DIF was small. The impact of DIF on the scale level (DTF) was negligible.

We employed a rigorous method to assess the dimensionality of the 4DSQ scales, using Yen’s Q3 [40]. In combination with Christensen’s method to determine critical Q3-values [42], the method turned out to be more sensitive to multidimensionality than more traditional fit statistics like the RMSEA. Our results indicate that the 4DSQ scales are essentially unidimensional, i.e., unidimensional enough to be treated as unidimensional in the context of IRT. Interestingly, the 4DSQ scales appeared to be slightly more unidimensional in the Web group than in the P&P group as evidenced by the slightly greater variance explained by the general factors (Online Resource 2). Apparently, the Web-based 4DSQ performs somewhat better, but certainly not worse, than the original P&P version.

DIF analysis is often concerned with inherently different groups (e.g., gender), in which case randomization is not feasible. In theory, DIF analysis is not hindered by group differences in trait levels because comparisons are matched at the trait level so that DIF only emerges when there is measurement bias rather than genuine trait differences. However, when groups differ in more respects than the trait level and the factor of interest (e.g., gender), the interpretation of the source of possible DIF may become problematic. In other words, when DIF is found, any aspect (other than trait level) in which the groups differ can potentially be the source of that DIF. Applied to the field of mode of administration equivalence research, no need for randomization may be an advantage of DIF analysis, but potential problems in the interpretation of DIF constitute a disadvantage. To avoid interpretation problems in this particular field, subjects can be randomly allocated to different mode of administration groups. But then data must specifically be collected for the evaluation of measurement equivalence, whereas data from different groups are often available ‘on the shelf,’ which is much cheaper.

In conclusion, using IRT-based DIF and DTF analysis to examine measurement equivalence across Web-based and P&P versions of the 4DSQ yielded few items with negligible DIF. Results obtained with the Web-based 4DSQ are equivalent to results obtained using the original P&P version of the questionnaire.

## Electronic supplementary material

Below is the link to the electronic supplementary material.

Supplementary material 1 (DOCX 21 KB)

Supplementary material 2 (DOCX 30 KB)

Supplementary material 3 (DOCX 20 KB)

## References

[1] van Gelder MM, Bretveld RW, Roeleveld N (2010). Web-based questionnaires: the future in epidemiology?. American Journal of Epidemiology.

[2] Coons SJ, Gwaltney CJ, Hays RD, Lundy JJ, Sloan JA, Revicki DA, Lenderking WR, Cella D, Basch E, on behalf of the ISPOR ePRO Task Force (2009). Recommendations on evidence needed to support measurement equivalence between electronic and paper-based patient-reported outcome (PRO) measures: ISPOR ePRO Good Research Practices Task Force report. Value in Health.

[3] Gwaltney CJ, Shields AL, Shiffman S (2008). Equivalence of electronic and paper-and-pencil administration of patient-reported outcome measures: A meta-analytic review. Value in Health.

[4] Campbell N, Ali F, Finlay AY, Salek SS (2015). Equivalence of electronic and paper-based patient-reported outcome measures. Quality of Life Research.

[5] Muehlhausen W, Doll H, Quadri N, Fordham B, O’Donohoe P, Dogar N, Wild DJ (2015). Equivalence of electronic and paper administration of patient-reported outcome measures: A systematic review and meta-analysis of studies conducted between 2007 and 2013. Health and Quality of Life Outcomes.

[6] Rutherford C, Costa D, Mercieca-Bebber R, Rice H, Gabb L, King M (2016). Mode of administration does not cause bias in patient-reported outcome results: A meta-analysis. Quality of Life Research.

[7] Twiss J, McKenna SP, Graham J, Swetz K, Sloan J, Gomberg-Maitland M (2016). Applying Rasch analysis to evaluate measurement equivalence of different administration formats of the Activity Limitation scale of the Cambridge Pulmonary Hypertension Outcome Review (CAMPHOR). Health and Quality of Life Outcomes.

[8] Ferrando PJ, Lorenzo-Seva U (2005). IRT-related factor analytic procedures for testing the equivalence of paper-and-pencil and Internet-administered questionnaires. Psychological Methods.

[9] Teresi JA, Fleishman JA (2007). Differential item functioning and health assessment. Quality of Life Research.

[10] Einarsdóttir S, Rounds J (2009). Gender bias and construct validity in vocational interest measurement: Differential item functioning in the Strong Interest Inventory. Journal of Vocational Behavior.

[11] Petersen MA, Groenvold M, Bjorner JB, Aaronson N, Conroy T, Cull A, Fayers P, Hjermstad M, Sprangers M, Sullivan M, For the European Organisation for Research and Treatment of Cancer Quality of Life Group (2003). Use of differential item functioning analysis to assess the equivalence of translations of a questionnaire. Quality of Life Research.

[12] Zumbo BD (2007). Three generations of DIF analyses: Considering where it has been, where it is now, and where it is going. Language Assessment Quarterly.

[13] Donovan MA, Drasgow F, Probst TM (2000). Does computerizing paper-and-pencil job attitude scales make a difference? New IRT analyses offer insight. Journal of Applied Psychology.

[14] Whitaker BG, McKinney JL (2007). Assessing the measurement invariance of latent job satisfaction ratings across survey administration modes for respondent subgroups: A MIMIC modeling approach. Behavior Research Methods.

[15] Swartz RJ, de Moor C, Cook KF, Fouladi RT, Basen-Engquist K, Eng C, Taylor CCL (2007). Mode effects in the center for epidemiologic studies depression (CES-D) scale: personal digital assistant vs. paper and pencil administration. Quality of Life Research.

[16] Michaelides MP (2008). An illustration of a Mantel-Haenszel procedure to flag misbehaving common items in test equating. Practical Assessment, Research & Evaluation.

[17] Dorans NJ, Schmitt AP, Bleistein CA (1992). The standardization approach to assessing comprehensive differential item functioning. Journal of Educational Measurement.

[18] Zumbo BD (1999). A handbook on the theory and methods of differential item functioning (DIF): Logistic regression modeling as a unitary framework for binary and likert-type (ordinal) item scores.

[19] Tay L, Meade AW, Cao MY (2015). An overview and practical guide to IRT measurement equivalence analysis. Organizational Research Methods.

[20] Gregorich SE (2006). Do self-report instruments allow meaningful comparisons across diverse population groups? Testing measurement invariance using the confirmatory factor analysis framework. Medical Care.

[21] Terluin B, van Marwijk HWJ, Adèr HJ, de Vet HCW, Penninx BWJH, Hermens MLM, van Boeijen CA, van Balkom AJLM, van der Klink JJL, Stalman WAB (2006). The Four-Dimensional Symptom Questionnaire (4DSQ): a validation study of a multidimensional self-report questionnaire to assess distress, depression, anxiety and somatization. BMC Psychiatry.

[22] Ridner SH (2004). Psychological distress: concept analysis. Journal of Advanced Nursing.

[23] Snaith RP (1987). The concepts of mild depression. British Journal of Psychiatry.

[24] Beck AT, Rush AJ, Shaw BF, Emery G (1979). Cognitive therapy of depression.

[25] Terluin B, Brouwers EPM, van Marwijk HWJ, Verhaak PFM, van der Horst HE (2009). Detecting depressive and anxiety disorders in distressed patients in primary care; comparative diagnostic accuracy of the Four-Dimensional Symptom Questionnaire (4DSQ) and the Hospital Anxiety and Depression Scale (HADS). BMC Family Practice.

[26] van Avendonk MJP, Hassink-Franke LJA, Terluin B, van Marwijk HWJ, Wiersma T, Burgers JS (2012). NHG-Standaard Angst (tweede herziening) [Summarisation of the NHG practice guideline ‘Anxiety’]. Nederlands Tijdschrift voor Geneeskunde.

[27] Terluin B, Oosterbaan DB, Brouwers EPM, van Straten A, van de Ven PM, Langerak W, van Marwijk HWJ (2014). To what extent does the anxiety scale of the Four-Dimensional Symptom Questionnaire (4DSQ) detect specific types of anxiety disorder in primary care? A psychometric study. BMC Psychiatry.

[28] Clarke DM, Smith GC (2000). Somatisation: what is it?. Australian Family Physician.

[29] de Vroege L, Emons WHM, Sijtsma K, Hoedeman R, van der Feltz-Cornelis CM (2015). Validation of the 4DSQ somatization subscale in the occupational health care setting as a screener. Journal of Occupational Rehabilitation.

[30] Terluin B, van Rhenen W, Schaufeli WB, de Haan M (2004). The Four-Dimensional Symptom Questionnaire (4DSQ): measuring distress and other mental health problems in a working population. Work Stress.

[31] van Ginkel JR, van der Ark LA (2005). SPSS syntax for missing value imputation in test and questionnaire data. Applied Psychological Measurement.

[32] Sijtsma K, van der Ark LA (2003). Investigation and treatment of missing item scores in test and questionnaire data. Multivariate Behavioral Research.

[33] Reeve BB, Hays RD, Bjorner JB, Cook KF, Crane PK, Teresi JA, Thissen D, Revicki DA, Weiss DJ, Hambleton RK, Liu HH, Gershon R, Reise SP, Lai JS, Cella D (2007). Psychometric evaluation and calibration of health-related quality of life item banks - Plans for the patient-reported outcomes measurement information system (PROMIS). Medical Care.

[34] Reise SP, Morizot J, Hays RD (2007). The role of the bifactor model in resolving dimensionality issues in health outcomes measures. Quality of Life Research.

[35] Stout WF (1990). A new item response theory modeling approach with applications to unidimensional assessment and ability estimation. Psychometrika.

[36] Chen WH, Thissen D (1997). Local dependence indexes for item pairs: Using item response theory. Journal of Educational and Behavioral Statistics.

[37] Cai L, Hansen M (2013). Limited-information goodness-of-fit testing of hierarchical item factor models. British Journal of Mathematical & Statistical Psychology.

[38] Hu L, Bentler PM (1999). Cutoff criteria for fit indexes in covariance structure analysis: Conventional criteria versus new alternatives. Structural Equation Modeling.

[39] Glöckner-Rist A, Hoijtink H (2003). The best of both worlds: Factor analysis of dichotomous data using item response theory and structural equation modeling. Structural Equation Modeling.

[40] Yen WM (1984). Effects of local item dependence on the fit and equating performance of the 3-parameter logistic model. Applied Psychological Measurement.

[41] Zenisky AL, Hambleton RK, Sireci SG (2003). Effects of local item dependence on the validity of IRT item, test, and ability statistics.

[42] Christensen KB, Makransky G, Horton M (2016). Critical values for Yen’s Q3: Identification of local dependence in the Rasch model using residual correlations. Applied Psychological Measurement.

[43] Reise SP (2012). The rediscovery of bifactor measurement models. Multivariate Behavioral Research.

[44] Rodriguez A, Reise SP, Haviland MG (2016). Applying bifactor statistical indices in the evaluation of psychological measures. Journal of Personality Assessment.

[45] Reise SP, Scheines R, Widaman KF, Haviland MG (2012). Multidimensionality and structural coefficient bias in structural equation modeling: A bifactor perspective. Educational and Psychological Measurement.

[46] Bonifay WE, Reise SP, Scheines R, Meijer RR (2015). When are multidimensional data unidimensional enough for structural equation modeling? An evaluation of the DETECT multidimensionality index. Structural Equation Modeling: A Multidisciplinary Journal.

[47] Cohen J (1977). Statistical power analysis for the behavioral sciences.

[48] Hays RD, Morales LS, Reise SP (2000). Item response theory and health outcomes measurement in the 21st century. Medical Care.

[49] Nguyen TH, Han HR, Kim MT, Chan KS (2014). An introduction to item response theory for patient-reported outcome measurement. Patient.

[50] Langer MM (2008). A reexamination of Lord’s Wald test for differential item functioning using item response theory and modern error estimation.

[51] Woods CM, Cai L, Wang M (2012). The Langer-improved Wald test for DIF testing with multiple groups. Educational and Psychological Measurement.

[52] Meade AW (2010). A taxonomy of measurement invariance effect size indices. Journal of Applied Psychology.

[53] Chalmers RP (2012). mirt: A multidimensional item response theory package for the R environment. Journal of Statistical Software.

[54] Chalmers, R. P. (2017). Package ‘mirt’, version 1.22. https://cran.r-project.org/web/packages/mirt/mirt.pdf.

[55] R Core Team (2016). R: A language and environment for statistical computing.

