# Supplementary material for: Assessing the equivalence of Web-based and paper-and-pencil questionnaires using differential item and test functioning (DIF and DTF) analysis: a case of the Four-Dimensional Symptom Questionnaire (4DSQ)
Source: Qual Life Res. 2018 Feb 21;27(5):1191–200. doi: 10.1007/s11136-018-1816-5 (PMC5891556; doi:10.1007/s11136-018-1816-5)
Supplement: Supplementary file 1 — Supplementary material 1 (DOCX 21 KB) [file 11136_2018_1816_MOESM1_ESM.docx]

Online Resource 1

**Four-Dimensional Symptom Questionnaire (4DSQ) – items and scales**

Article Title:

**Assessing the equivalence of Web-based and paper-and-pencil questionnaires using differential item and test functioning (DIF and DTF) analysis: A case of the Four-Dimensional Symptom Questionnaire (4DSQ)**

Journal name:

**Quality of Life Research**

Author names:

Berend Terluin^1^, Evelien P. M. Brouwers^2^, Miquelle A. G. Marchand^3^, Henrica C. W. de Vet^4^

*^1^ Department of General Practice and Elderly Care Medicine, Amsterdam Public Health research institute, VU University Medical Center, Amsterdam, the Netherlands*

*^2^ Scientific Center for Care and Welfare (Tranzo), Tilburg University, Tilburg, the Netherlands*

*^3^ CentERdata Institute for Data Collection and Research, Tilburg University, Tilburg, the Netherlands*

*^4^ Department of Epidemiology & Biostatistics, Amsterdam Public Health research institute, VU University Medical Center, Amsterdam, the Netherlands*

Phone: +31 20 4448199

Fax: +31 20 4448195

Email: [b.terluin@vumc.nl](mailto:b.terluin@vumc.nl)

ORCID (B.Terluin): 0000-0002-8944-5238

**Four-Dimensional Symptom Questionnaire (4DSQ) – items and scales**

**Instruction:**

The following is a list of questions about various complaints and symptoms you may have. Each question refers to the complaints and symptoms that you had **in the past week (the past 7 days, including today)**. Complaints you had before then, but no longer had during the past week, do not count. Please indicate for each complaint how often you noticed that you had it in the past week by putting an “X” in the box under the answer that is most appropriate.

| **Nr.** | **Item** | | **Scale*** |
| --- | --- | --- | --- |
| **During the past week, did you suffer from:** | | |  |
| 1 | | dizziness or feeling light-headed? | SOM |
| 2 | | painful muscles? | SOM |
| 3 | | fainting? | SOM |
| 4 | | neck pain? | SOM |
| 5 | | back pain? | SOM |
| 6 | | excessive sweating? | SOM |
| 7 | | palpitations? | SOM |
| 8 | | headache? | SOM |
| 9 | | a bloated feeling in the abdomen? | SOM |
| 10 | | blurred vision or spots in front of your eyes? | SOM |
| 11 | | shortness of breath? | SOM |
| 12 | | nausea or an upset stomach? | SOM |
|  | |  |  |
| **During the past week, did you suffer from:** | | |  |
| 13 | | pain in the abdomen or stomach area? | SOM |
| 14 | | tingling in the fingers? | SOM |
| 15 | | pressure or a tight feeling in the chest? | SOM |
| 16 | | pain in the chest? | SOM |
| 17 | | feeling down or depressed? | DIS |
| 18 | | sudden fright for no reason? | ANX |
| 19 | | worry? | DIS |
| 20 | | disturbed sleep? | DIS |
| 21 | | a vague feeling of fear? | ANX |
| 22 | | lack of energy? | DIS |
| 23 | | trembling when with other people? | ANX |
| 24 | | anxiety or panic attacks? | ANX |
|  | | |  |
| **During the past week, did you feel:** | | |  |
| 25 | | tense? | DIS |
| 26 | | easily irritated? | DIS |
| 27 | | frightened? | ANX |
|  | | |  |
| **During the past week, did you feel:** | | |  |
| 28 | | that everything is meaningless? | DEP |
| 29 | | that you just can't do anything anymore? | DIS |
| 30 | | that life is not worth while? | DEP |
| 31 | | that you can no longer take any interest in the people and things around you? | DIS |
| 32 | | that you can't cope anymore? | DIS |
| 33 | | that you would be better off if you were dead? | DEP |
| 34 | | that you can't enjoy anything anymore? | DEP |
| 35 | | that there is no escape from your situation? | DEP |
| 36 | | that you can't face it anymore? | DIS |
|  | |  |  |
| **During the past week, did you:** | | |  |
| 37 | | no longer feel like doing anything? | DIS |
| 38 | | have difficulty in thinking clearly? | DIS |
| 39 | | have difficulty in getting to sleep? | DIS |
| 40 | | have any fear of going out of the house alone? | ANX |
|  | |  |  |
| **During the past week:** | | |  |
| 41 | | did you easily become emotional? | DIS |
| 42 | | were you afraid of anything when there was really no need for you to be afraid?  *(for instance animals, heights, small rooms)* | ANX |
| 43 | | were you afraid to travel on buses, streetcars/ trams, subways or trains? | ANX |
| 44 | | were you afraid of becoming embarrassed when with other people? | ANX |
| 45 | | did you ever feel as if you were being threatened by unknown danger? | ANX |
| 46 | | did you ever think "I wish I was dead"? | DEP |
| 47 | | did you ever have fleeting images of any upsetting event(s) that you have experienced? | DIS |
| 48 | | did you ever have to do your best to put aside thoughts about any upsetting event(s)? | DIS |
| 49 | | did you have to avoid certain places because they frightened you? | ANX |
| 50 | | did you have to repeat some actions a number of times before you could do something else? | ANX |

* Scale: DIS = distress, DEP = depression, ANX = anxiety, SOM = somatization

| **Scoring:** | no | sometimes | regularly | often | very often or constantly |
| --- | --- | --- | --- | --- | --- |
| **Coding:** | 0 | 1 | 2 | 2 | 2 |
