# Supplementary material for: Assessing the equivalence of Web-based and paper-and-pencil questionnaires using differential item and test functioning (DIF and DTF) analysis: a case of the Four-Dimensional Symptom Questionnaire (4DSQ)
Source: Qual Life Res. 2018 Feb 21;27(5):1191–200. doi: 10.1007/s11136-018-1816-5 (PMC5891556; doi:10.1007/s11136-018-1816-5)
Supplement: Supplementary file 2 — Supplementary material 2 (DOCX 30 KB) [file 11136_2018_1816_MOESM2_ESM.docx]

Online Resource 2

**Standardized factor loadings of the IRT models**

Article Title:

**Assessing the equivalence of Web-based and paper-and-pencil questionnaires using differential item and test functioning (DIF and DTF) analysis: A case of the Four-Dimensional Symptom Questionnaire (4#)**

Journal name:

**Quality of Life Research**

Author names:

Berend Terluin^1^, Evelien P. M. Brouwers^2^, Miquelle A. G. Marchand^3^, Henrica C. W. de Vet^4^

*^1^ Department of General Practice and Elderly Care Medicine, Amsterdam Public Health research institute, VU University Medical Center, Amsterdam, the Netherlands*

*^2^ Scientific Center for Care and Welfare (Tranzo), Tilburg University, Tilburg, the Netherlands*

*^3^ CentERdata Institute for Data Collection and Research, Tilburg University, Tilburg, the Netherlands*

*^4^ Department of Epidemiology & Biostatistics, Amsterdam Public Health research institute, VU University Medical Center, Amsterdam, the Netherlands*

Phone: +31 20 4448199

Fax: +31 20 4448195

Email: [b.terluin@vumc.nl](mailto:b.terluin@vumc.nl)

ORCID (B.Terluin): 0000-0002-8944-5238

**Standardized factor loadings of the IRT models**

**Distress P&P**

| **Item** | **Unidimensional** | **Bifactor** | | | |
| --- | --- | --- | --- | --- | --- |
|  | **F1** | **G** | **S1** | **S2** | **S3** |
| #17 | 0.803 | 0.805 |  |  |  |
| #19 | 0.789 | 0.784 |  |  |  |
| #20 | 0.601 | 0.576 |  | 0.685 |  |
| #22 | 0.815 | 0.827 |  |  |  |
| #25 | 0.777 | 0.769 |  |  |  |
| #26 | 0.575 | 0.576 |  |  |  |
| #29 | 0.854 | 0.864 |  |  |  |
| #31 | 0.771 | 0.779 |  |  |  |
| #32 | 0.839 | 0.806 |  |  | 0.446 |
| #36 | 0.822 | 0.787 |  |  | 0.465 |
| #37 | 0.882 | 0.891 |  |  |  |
| #38 | 0.739 | 0.734 |  |  |  |
| #39 | 0.494 | 0.478 |  | 0.736 |  |
| #41 | 0.656 | 0.645 |  |  |  |
| #47 | 0.461 | 0.429 | 0.800 |  |  |
| #48 | 0.531 | 0.497 | 0.769 |  |  |
| *Proportion Variance* | *0.526* | *0.514* | *0.077* | *0.063* | *0.026* |

**Distress Web**

| **Item** | **Unidimensional** | **Bifactor** | | | |
| --- | --- | --- | --- | --- | --- |
|  | **F1** | **G** | **S1** | **S2** | **S3** |
| #17 | 0.813 | 0.818 |  |  |  |
| #19 | 0.821 | 0.819 |  |  |  |
| #20 | 0.642 | 0.613 |  | 0.642 |  |
| #22 | 0.823 | 0.832 |  |  |  |
| #25 | 0.816 | 0.809 |  |  |  |
| #26 | 0.694 | 0.696 |  |  |  |
| #29 | 0.857 | 0.858 |  |  |  |
| #31 | 0.816 | 0.822 |  |  |  |
| #32 | 0.874 | 0.843 |  |  | 0.407 |
| #36 | 0.888 | 0.857 |  |  | 0.391 |
| #37 | 0.886 | 0.891 |  |  |  |
| #38 | 0.758 | 0.755 |  |  |  |
| #39 | 0.549 | 0.536 |  | 0.686 |  |
| #41 | 0.697 | 0.688 |  |  |  |
| #47 | 0.504 | 0.476 | 0.800 |  |  |
| #48 | 0.569 | 0.535 | 0.769 |  |  |
| *Proportion Variance* | *0.578* | *0.565* | *0.077* | *0.055* | *0.020* |

**Depression P&P**

| **Item** | **Unidimensional** | **Bifactor** | | | |
| --- | --- | --- | --- | --- | --- |
|  | **F1** | **G** | **S1** |  |  |
| #28 | 0.828 | 0.827 |  |  |  |
| #30 | 0.939 | 0.939 |  |  |  |
| #33 | 0.966 | 0.969 |  |  |  |
| #34 | 0.775 | 0.758 | 0.359 |  |  |
| #35 | 0.716 | 0.695 | 0.396 |  |  |
| #46 | 0.954 | 0.956 |  |  |  |
| *Proportion Variance* | *0.754* | *0.746* | *0.048* |  |  |

**Depression Web**

| **Item** | **Unidimensional** | **Bifactor** | | | |
| --- | --- | --- | --- | --- | --- |
|  | F1 | G | S1 |  |  |
| #28 | 0.896 | 0.907 |  |  |  |
| #30 | 0.961 | 0.967 |  |  |  |
| #33 | 0.950 | 0.892 | 0.411 |  |  |
| #34 | 0.832 | 0.842 |  |  |  |
| #35 | 0.815 | 0.819 |  |  |  |
| #46 | 0.933 | 0.860 | 0.465 |  |  |
| *Proportion Variance* | *0.809* | *0.779* | *0.064* |  |  |

**Anxiety P&P**

| **Item** | **Unidimensional** | **Bifactor** | | | |
| --- | --- | --- | --- | --- | --- |
|  | **F1** | **G** | **S1** | **S2** |  |
| #18 | 0.650 | 0.648 |  |  |  |
| #21 | 0.895 | 0.904 |  |  |  |
| #23 | 0.649 | 0.635 |  | 0.388 |  |
| #24 | 0.847 | 0.857 |  |  |  |
| #27 | 0.897 | 0.909 |  |  |  |
| #40 | 0.744 | 0.688 | 0.420 |  |  |
| #42 | 0.651 | 0.606 | 0.374 |  |  |
| #43 | 0.703 | 0.591 | 0.656 |  |  |
| #44 | 0.469 | 0.448 |  | 0.450 |  |
| #45 | 0.676 | 0.673 |  |  |  |
| #49 | 0.717 | 0.673 | 0.357 |  |  |
| #50 | 0.478 | 0.474 |  |  |  |
| *Proportion Variance* | *0.505* | *0.477* | *0.073* | *0.029* |  |

**Anxiety Web**

| **Item** | **Unidimensional** | **Bifactor** | | | |
| --- | --- | --- | --- | --- | --- |
|  | **F1** | **G** | **S1** |  |  |
| #18 | 0.742 | 0.742 |  |  |  |
| #21 | 0.901 | 0.909 |  |  |  |
| #23 | 0.683 | 0.684 |  |  |  |
| #24 | 0.845 | 0.851 |  |  |  |
| #27 | 0.917 | 0.923 |  |  |  |
| #40 | 0.805 | 0.764 | 0.369 |  |  |
| #42 | 0.753 | 0.722 | 0.334 |  |  |
| #43 | 0.810 | 0.744 | 0.525 |  |  |
| #44 | 0.583 | 0.575 |  |  |  |
| #45 | 0.723 | 0.719 |  |  |  |
| #49 | 0.803 | 0.796 |  |  |  |
| #50 | 0.511 | 0.506 |  |  |  |
| *Proportion Variance* | *0.585* | *0.568* | *0.044* |  |  |

**Somatization P&P**

| **Item** | **Unidimensional** | **Bifactor** | | | |
| --- | --- | --- | --- | --- | --- |
|  | **F1** | **G** | **S1** | **S2** | **S3** |
| #01 | 0.649 | 0.719 |  |  |  |
| #02 | 0.542 | 0.485 |  | 0.625 |  |
| #03 | 0.559 | 0.583 |  |  |  |
| #04 | 0.593 | 0.548 |  | 0.531 |  |
| #05 | 0.549 | 0.490 |  | 0.559 |  |
| #06 | 0.494 | 0.517 |  |  |  |
| #07 | 0.581 | 0.545 |  |  | 0.379 |
| #08 | 0.573 | 0.604 |  |  |  |
| #09 | 0.563 | 0.496 | 0.483 |  |  |
| #10 | 0.613 | 0.677 |  |  |  |
| #11 | 0.652 | 0.607 |  |  | 0.325 |
| #12 | 0.593 | 0.541 | 0.581 |  |  |
| #13 | 0.611 | 0.522 | 0.723 |  |  |
| #14 | 0.516 | 0.530 |  |  |  |
| #15 | 0.685 | 0.586 |  |  | 0.661 |
| #16 | 0.698 | 0.567 |  |  | 0.672 |
| *Proportion Variance* | *0.353* | *0.322* | *0.068* | *0.062* | *0.071* |

**Somatization Web**

| **Item** | **Unidimensional** | **Bifactor** | | | |
| --- | --- | --- | --- | --- | --- |
|  | **F1** | **G** | **S1** | **S2** | **S3** |
| #01 | 0.648 | 0.687 |  |  |  |
| #02 | 0.608 | 0.536 |  | 0.649 |  |
| #03 | 0.558 | 0.574 |  |  |  |
| #04 | 0.594 | 0.520 |  | 0.610 |  |
| #05 | 0.606 | 0.526 |  | 0.582 |  |
| #06 | 0.570 | 0.588 |  |  |  |
| #07 | 0.652 | 0.686 |  |  |  |
| #08 | 0.594 | 0.593 |  |  |  |
| #09 | 0.594 | 0.525 | 0.487 |  |  |
| #10 | 0.608 | 0.644 |  |  |  |
| #11 | 0.746 | 0.769 |  |  |  |
| #12 | 0.718 | 0.664 | 0.509 |  |  |
| #13 | 0.669 | 0.579 | 0.681 |  |  |
| #14 | 0.582 | 0.593 |  |  |  |
| #15 | 0.733 | 0.727 |  |  | 0.581 |
| #16 | 0.731 | 0.681 |  |  | 0.620 |
| *Proportion Variance* | *0.411* | *0.388* | *0.060* | *0.071* | *0.045* |
