# Supplementary material for: Assessing the equivalence of Web-based and paper-and-pencil questionnaires using differential item and test functioning (DIF and DTF) analysis: a case of the Four-Dimensional Symptom Questionnaire (4DSQ)
Source: Qual Life Res. 2018 Feb 21;27(5):1191–200. doi: 10.1007/s11136-018-1816-5 (PMC5891556; doi:10.1007/s11136-018-1816-5)
Supplement: Supplementary file 3 — Supplementary material 3 (DOCX 20 KB) [file 11136_2018_1816_MOESM3_ESM.docx]

Online Resource 3

**Item parameters by group**

Article Title:

**Assessing the equivalence of Web-based and paper-and-pencil questionnaires using differential item and test functioning (DIF and DTF) analysis: A case of the Four-Dimensional Symptom Questionnaire (4DSQ)**

Journal name:

**Quality of Life Research**

Author names:

Berend Terluin^1^, Evelien P. M. Brouwers^2^, Miquelle A. G. Marchand^3^, Henrica C. W. de Vet^4^

*^1^ Department of General Practice and Elderly Care Medicine, Amsterdam Public Health research institute, VU University Medical Center, Amsterdam, the Netherlands*

*^2^ Scientific Center for Care and Welfare (Tranzo), Tilburg University, Tilburg, the Netherlands*

*^3^ CentERdata Institute for Data Collection and Research, Tilburg University, Tilburg, the Netherlands*

*^4^ Department of Epidemiology & Biostatistics, Amsterdam Public Health research institute, VU University Medical Center, Amsterdam, the Netherlands*

Phone: +31 20 4448199

Fax: +31 20 4448195

Email: [b.terluin@vumc.nl](mailto:b.terluin@vumc.nl)

ORCID (B.Terluin): 0000-0002-8944-5238

**Item parameters by group**

| **Scale/Items** | **Web group** | | | **P&P group** | | |
| --- | --- | --- | --- | --- | --- | --- |
| **Distress** | ***a*** | ***b1*** | ***b2*** | ***a*** | ***b1*** | ***b2*** |
| #17 | 2.200 | -1.161 | 0.115 | 2.281 | -1.223 | -0.213 |
| #19 | 2.195 | -2.135 | -1.009 | 2.195 | -2.135 | -1.009 |
| #20 | 1.277 | -1.646 | -0.458 | 1.277 | -1.646 | -0.458 |
| #22 | 2.340 | -0.927 | 0.053 | 2.340 | -0.927 | 0.053 |
| #25 | 2.125 | -1.715 | -0.527 | 2.125 | -1.715 | -0.527 |
| #26 | 1.286 | -1.603 | -0.105 | 1.286 | -1.603 | -0.105 |
| #29 | 2.724 | -0.458 | 0.356 | 2.724 | -0.458 | 0.356 |
| #31 | 2.103 | -0.268 | 0.798 | 2.103 | -0.268 | 0.798 |
| #32 | 2.679 | -0.457 | 0.464 | 2.679 | -0.457 | 0.464 |
| #36 | 2.607 | -0.418 | 0.531 | 2.607 | -0.418 | 0.531 |
| #37 | 3.117 | -0.497 | 0.367 | 3.117 | -0.497 | 0.367 |
| #38 | 1.830 | -0.786 | 0.371 | 1.830 | -0.786 | 0.371 |
| #39 | 0.983 | -0.905 | 0.371 | 0.983 | -0.905 | 0.371 |
| #41 | 1.492 | -1.379 | -0.228 | 1.492 | -1.379 | -0.228 |
| #47 | 0.910 | -0.689 | 1.033 | 0.884 | -0.929 | 0.540 |
| #48 | 1.080 | -0.286 | 1.057 | 1.066 | -0.493 | 0.591 |
| **Depression** |  |  |  |  |  |  |
| #28 | 2.708 | -0.195 | 0.616 | 2.708 | -0.195 | 0.616 |
| #30 | 4.887 | 0.312 | 1.037 | 4.887 | 0.312 | 1.037 |
| #33 | 5.869 | 0.666 | 1.445 | 5.869 | 0.666 | 1.445 |
| #34 | 2.169 | -0.270 | 0.646 | 2.169 | -0.270 | 0.646 |
| #35 | 1.880 | -0.037 | 0.894 | 1.880 | -0.037 | 0.894 |
| #46 | 5.018 | 0.763 | 1.533 | 5.018 | 0.763 | 1.533 |
| **Anxiety** |  |  |  |  |  |  |
| #18 | 1.534 | 0.406 | 1.384 | 1.534 | 0.406 | 1.384 |
| #21 | 3.307 | -0.366 | 0.330 | 3.307 | -0.366 | 0.330 |
| #23 | 1.446 | 0.997 | 1.988 | 1.446 | 0.997 | 1.988 |
| #24 | 2.596 | 0.396 | 1.177 | 2.596 | 0.396 | 1.177 |
| #27 | 3.437 | -0.224 | 0.561 | 3.437 | -0.224 | 0.561 |
| #40 | 1.961 | 1.261 | 1.902 | 1.961 | 1.261 | 1.902 |
| #42 | 1.562 | 1.049 | 1.811 | 1.562 | 1.049 | 1.811 |
| #43 | 1.834 | 1.749 | 2.243 | 1.834 | 1.749 | 2.243 |
| #44 | 0.968 | 0.528 | 1.967 | 0.968 | 0.528 | 1.967 |
| #45 | 1.578 | 1.391 | 2.194 | 1.578 | 1.391 | 2.194 |
| #49 | 1.846 | 1.240 | 1.940 | 1.846 | 1.240 | 1.940 |
| #50 | 0.913 | 1.461 | 2.791 | 0.913 | 1.461 | 2.791 |
| **Somatization** |  |  |  |  |  |  |
| #01 | 1.420 | -0.203 | 1.243 | 1.420 | -0.203 | 1.243 |
| #02 | 1.160 | -0.524 | 0.614 | 1.160 | -0.524 | 0.614 |
| #03 | 1.149 | 3.517 | 4.800 | 1.149 | 3.517 | 4.800 |
| #04 | 1.251 | -0.281 | 0.671 | 1.251 | -0.281 | 0.671 |
| #05 | 1.173 | -0.394 | 0.784 | 1.173 | -0.394 | 0.784 |
| #06 | 1.010 | 0.017 | 1.333 | 1.010 | 0.017 | 1.333 |
| #07 | 1.271 | 0.252 | 1.546 | 1.271 | 0.252 | 1.546 |
| #08 | 1.196 | -0.650 | 0.703 | 1.196 | -0.650 | 0.703 |
| #09 | 1.171 | 0.063 | 1.262 | 1.171 | 0.063 | 1.262 |
| #10 | 1.289 | 0.466 | 1.718 | 1.289 | 0.466 | 1.718 |
| #11 | 1.552 | 0.694 | 1.674 | 1.552 | 0.694 | 1.674 |
| #12 | 1.713 | 0.164 | 1.361 | 1.242 | -0.054 | 1.091 |
| #13 | 1.494 | 0.447 | 1.687 | 1.301 | 0.332 | 1.388 |
| #14 | 1.073 | 0.880 | 1.979 | 1.073 | 0.880 | 1.979 |
| #15 | 1.639 | 0.542 | 1.555 | 1.639 | 0.542 | 1.555 |
| #16 | 1.659 | 1.232 | 2.171 | 1.659 | 1.232 | 2.171 |
